# Targeting of EIF4EBP1 by miR‐99a‐3p affects the functions of B lymphocytes via autophagy and aggravates SLE disease progression

**DOI:** 10.1111/jcmm.16991

**Published:** 2021-10-20

**Authors:** Meng Yang, Binbin Yang, Danqi Deng

**Affiliations:** ^1^ Department of Dermatology The Second Affiliated Hospital of Kunming Medical University Kunming Yunnan China; ^2^ Department of Dermatology The Third Affiliated Hospital of Guangxi Medical University Nanning Guangxi China

**Keywords:** autophagy, B lymphocytes, EIF4EBP1, miR‐99a‐3p, systemic lupus erythematosus

## Abstract

Excessive activation of immune cells plays a key role in the pathogenesis of systemic lupus erythematosus (SLE). The regulation of immune cells by miRNAs is a research hotspot. In this study, second‐generation high‐throughput sequencing revealed a reduction in miR‐99a‐3p expression in patients with SLE; however, the specific mechanism underlying this phenomenon remains unclear. After transfection with an miR‐99a‐3p agomir, the proliferation of Ball‐1 cells decreased and the levels of their apoptosis increased. The opposite effects were observed in cells transfected with the miR‐99a‐3p antagomir. Luciferase reporter assay indicated that miR‐99a‐3p directly targeted EIF4EBP1. Rescue experiments confirmed the proposed interaction between miR‐99a‐3p and EIF4EBP1. *In vitro*, *in vivo* and clinical investigations further confirmed that the miR‐99a‐3p agomir reduced the expression of EIF4EBP1, LC3B and LAMP‐2A. In the *in vivo* experiments, serum levels of anti‐nuclear antibodies, double‐stranded DNA, IgE, IgM, IL‐6, IL‐10 and B lymphocyte stimulator were higher in mice from the antagomir group than those in mice from the MRL/lpr group. Furthermore, the protein and mRNA levels of EIF4EBP1, LC3B and LAMP‐2A, the intensity of immunohistochemical staining of EIF4EBP1, LC3B and LAMP‐2A, the urinary protein levels, and the C3 immunofluorescence deposition increased in mice from the antagomir group. The upregulation of miR‐99a‐3p expression protected B cells from EIF4EBP1‐mediated autophagy, whilst the downregulation of miR‐99a‐3p expression induced autophagy via the EIF4EBP1‐mediated regulation of the autophagy signalling pathway in B cells isolated from individuals with SLE. Based on these results, miR‐99a‐3p and EIF4EBP1 may be considered potential targets for SLE treatment.

## 1 INTRODUCTION

Systemic lupus erythematosus (SLE) is a type of autoimmune‐mediated diffuse connective tissue disease that involves multiple systems throughout the body and is characterized by pathological inflammation.^1^ Genetic factors influence the clinical phenotype and progression of SLE, whilst environmental factors, such as ultraviolet rays, promote the onset of SLE,^2^ which play an important role in stimulating SLE.^3^ The age of the onset of SLE in individuals residing in high‐altitude areas is lower, and the disease course is shorter.^4^

Tibetans comprise the main ethnic group in the Yunnan Diqing Tibetan Autonomous Prefecture; they live in a high‐altitude, low‐oxygen, dry, very cold environments with strong ultraviolet radiation. They are relatively isolated and typically do not contribute to genetic exchange with the rest of the world. Thus, the epidemiological and transcriptomic characteristics of Tibetan patients with SLE deserve in‐depth and systematic investigations.

An increasing number of noncoding single‐stranded small RNAs called microRNAs (miRNAs) have been found to play an important role in the pathogenesis of SLE.^5^ Previous studies performed by our research group indicated that UVB may mediate the pathogenesis of SLE by decreasing miR‐125b‐5p expression in patients with SLE and increasing the expression of the target gene *UVRAG* and the degree of cellular autophagy.^6^

**FIGURE 1:**
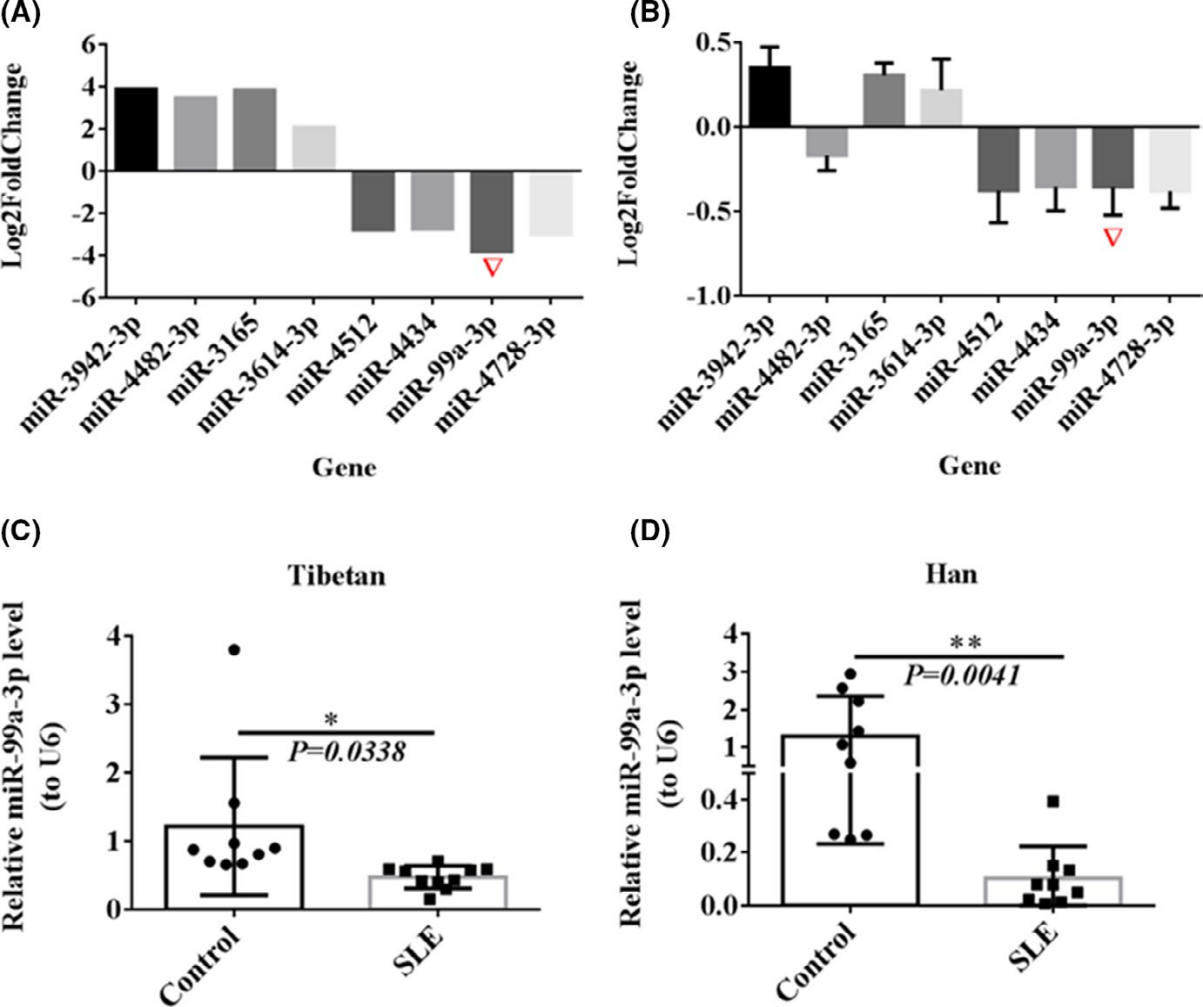
Differential verification of miR‐99a‐3p in SLE. (A) Screening of significantly differentially expressed miRNAs via second‐generation high‐throughput sequencing (B) RT‐qPCR verification of significantly differentially expressed miRNAs (C, D) RT‐qPCR detection of miR‐99a‐3p in PBMCs from Tibetan and Han SLE patients and Tibetan and Han healthy controls (*n* = 10)

We collected venous blood samples from 10 Tibetan patients with SLE and 10 healthy Tibetans. miRNA expression profiles were determined by second‐generation high‐throughput sequencing after RNA extraction (Figure 1A). We used RT‐qPCR to verify the sequencing results, and miR‐99a‐3p expression in Tibetan SLE patients was found to be significantly reduced (Figure 1B). The sequencing results were verified using RT‐qPCR, and miR‐99a‐3p expression was significantly decreased in Tibetan patients with SLE (Figure 1B).

miR‐99a‐3p is transcribed from the long arm of chromosome 21. miR‐99 is expressed at low levels in a variety of human malignancies. It participates in the progression of urinary tumours,^7^ squamous cell carcinoma,^8^ liver cancer^9^ and ovarian cancer^10^ and has a certain significance in the early diagnosis and determination of tumour stages.^11^

Pradhan and Tomankova were the first to demonstrate a reduction in miR‐99 expression in patients with SLE.^12^ Jin et al.^13^ observed the downregulation of miR‐99a expression in South Korean patients with SLE using miRNA PCR chip detection. Frangou et al.^14^ used cDNA microarrays to compare gene expression in the effector cells and target tissues of patients with SLE and control subjects and found that miR‐99a expression was decreased in PBMCs isolated from patients with SLE and was related to the regulation of the type I IFN pathway. However, thus far, no report has documented miR‐99a‐3p expression in Chinese patients with SLE.

Therefore, we explored the role of miR‐99a‐3p in the pathogenesis of SLE. This study attempts to elucidate the complex mechanisms underlying the pathogenesis of SLE and identifies new targets for SLE treatment.

## 2 MATERIALS AND METHODS

### 2.1 Patients and samples

From January to December 2020, we included ten SLE patients of Han ethnicity who were treated at our hospital's outpatient clinic. We contacted the People's Hospital of Diqing Tibetan Autonomous Prefecture in the Yunnan Province and collected blood samples from 10 Tibetan patients with SLE showing three generations of consanguinity without intermarriage, per the hospital's database. Both Tibetan and Han patients were diagnosed in accordance with the American Rheumatism Association 1997 classification of SLE and had not consumed chloroquine or hydroxychloroquine in the past three months. With the approval of the hospital ethics committee (PAR‐PJ‐2020–135), we collected 20 ml of peripheral venous blood from the patients and healthy controls. No significant differences in age or sex were observed between the two groups (*p* > 0.05, see Table S1).

### 2.2 Isolation of PBMCs and separation and culture of primary B cells

PBMCs were isolated using lymphocyte separation solution (LTS1077, Haoyang, Tianjin, China) containing 20 ml of the anticoagulant EDTA. RT‐qPCR and Western blotting were used to detect changes in gene and protein expression levels in PBMCs and the effects of interventions after culture.

A total of 1 ml of separated PBMCs (1 × 10^8^ cells) was placed in a 5‐ml test tube, and 200 µl of MagniSort^TM^ Human B Cell Enrichment Kit reagent (Thermo, Germany) was added. The samples were mixed well and incubated for 10 min. A total of 200 µl of MagniSort^TM^ Negative Selection Beads (Thermo, Germany) was added, followed by mixing and further incubation. The test tube was inserted into the MagniSort^TM^ magnetic pole (Thermo, Germany), and the precipitate was centrifuged to collect B lymphocytes (identification of B cells and culture, see Figure S1).

Frozen Ball‐1, Jurkat, THP‐1 and K562 cells (purchased from the Cell Bank of the Chinese Academy of Sciences) were collected from a liquid nitrogen tank, thawed in a water bath at 37°C and centrifuged; the supernatants were discarded. Next, the cells were resuspended in RPMI‐1640 (PM150110)+10% FBS (164210–500)+1% P/S (PB180120) and cultured.

### 2.3 Detection of miRNA/mRNA expression via RT‐qPCR

TRIzol™ Reagent (15596026, Invitrogen, USA) was used to extract the total RNA. A Transcriptor First Strand cDNA Synthesis Kit (Roche, Switzerland) was used for mRNA, and an All‐in‐One™ miRNA First‐Strand cDNA Synthesis for miRNA Kit 2.0 (GeneCopoeia, USA) was used for miRNA. A total of 1 µg of total RNA was used to synthesize the first‐strand cDNA templates for the mRNAs/miRNAs according to the manufacturers’ instructions. The reaction conditions for mRNA reverse transcription were as follows: 25°C for 5 min, 42°C for 45 min and 85°C for 5 min. The reaction conditions for miRNA reverse transcription were as follows: 37°C for 60 min and 85°C for 5 min. Reverse transcription was performed using a common PCR machine (Applied Biosystems 2720 Thermal Cycler, Thermo, Germany). BlazeTaq™ SYBR^®^ Green qPCR Mix 2.0 (GeneCopoeia, USA) was used for mRNA, and an All‐in‐One miRNA qRT‐PCR Detection Kit 2.0 (GeneCopoeia, USA) was used for miRNA, using cDNAs as the templates. GAPDH and U6 served as the internal references. The expression levels of the target genes were detected using the SYBR green method and a real‐time fluorescence quantitative PCR instrument (CFX96, Bio‐Rad, USA). The PCR amplification conditions were set as follows: 95°C for 10 min, followed by 40 cycles of 95°C for 10 sec, 60°C for 20 s and 72°C for 30 sec. The fluorescence was recorded, and the CT values were determined; the 2^−△△CT^ method was used to calculate the relative expression of the target genes. The relevant primers were synthesized by Beijing Kinco Xinye Biotechnology Co., Ltd.; the sequences of these primers are shown in Table S2.

### 2.4 Transfection of Ball‐1, Jurkat, THP‐1 and K562 cells with miR‐99a‐3p agomir and antagomir

The miR‐99a‐3p agomir (sense‐CAAGCUCGCUUCUAUGGGUCUG, antisense ‐CAGACCCAUAGAAG CGAGCUUG) and miR‐99a‐3p antagomir (sense‐CAGACCC AUAGAAGCGAGCUUG) were synthesized by RiboBio Co. (Guangzhou, China). When the cell confluence reached 80%, 2 µl of Lipo2000 transfection reagent (Thermo, Germany) was added for transient transfection. The medium was replaced after 6 h of transfection, and RT‐qPCR was performed after 48 h of culture.

### 2.5 CCK8 analysis of cell proliferation

Newly cultured Ball‐1, Jurkat, THP‐1 and K562 cell suspensions (100 µl/well) were seeded into 96‐well plates, and the four cell lines were transfected with the miR‐99a‐3p agomir and antagomir. A total of 10 µl of CCK8 reagent (Life iLab Biotech Co., Shanghai, China) was added to each well on days 1, 2, 3 and 4, followed by incubation for 2 h; the absorbance of the samples was measured at 450 nm using a microplate reader (BioTek, USA).

### 2.6 PI method to detect cell cycle distribution

A total of 250 µl of the suspension of each cell type (1 × 10^6^ cells) was mixed with 750 µl of precooled absolute ethanol; the samples were sealed and incubated overnight at −20°C for fixation. Next, 500 µl of PI staining solution (Becton, Dickinson and Company, USA) was added to obtain a final concentration of 65 µg/ml, followed by incubation at 37°C for 30 min. Subsequently, the cells were immediately analysed via flow cytometry (Thermo, Germany). The ModFit LT™ 4.1 software was used to analyse the cell cycle distribution.

### 2.7 FITC‐Annexin V staining to detect cell apoptosis

According to the instructions provided with the Annexin V FITC Apop Dtec Kit I apoptosis kit (556547, Shanghai, China), 300 µl of 1× binding buffer was added to the cells (1 × 10^6^ cells/ml), followed by the addition of 5 µl of Annexin V‐FITC and propidium iodide staining solution and incubation. The cells were analysed using an Attune NxT Flow Cytometer (Thermo, Germany).

### 2.8 Dual‐luciferase reporter assay

The target genes of miR‐99a‐3p were predicted, and EIF4EBP1, NCAPG, IKBKB and PRKCB were selected for luciferase detection. The wild‐type/mutant 3′‐UTR of the target genes was inserted into a luciferase pmirGLO vector by the Wuhan GeneCreate Company. The miRNA and plasmid (2 µg) were incubated together for 5 min, followed by the addition of 2 µl of Lipo2000 transfection reagent and further incubation in the culture medium. When the cell fusion degree reached 80%, 500 µl of the aforementioned transfection complex was added. The proteins were extracted and mixed with 100 µl of firefly luciferase substrate to measure the firefly luciferin value. *Renilla* luciferase substrate was added, and the activity of *Renilla* luciferase was then measured. The fluorescence value of the reporter plasmid in each well was obtained by dividing the *Renilla* fluorescence value in each well by the firefly fluorescein value.

### 2.9 Western blotting

For this experiment, 250 µl of RIPA lysis buffer (containing protease inhibitors, Thermo, Germany) was added to each group of cells, and a BCA protein quantification kit (P0010, Beyotime, China) was used to determine the protein concentration. Total protein (30 µg) was subjected to electrophoresis using SDS‐PAGE gels and transferred onto membranes, which were then blocked. The membranes were then incubated with primary antibodies against EIF4EBP1 (1:1000; GTX133182; GeneTex, USA), LAMP‐2A (1:1000; EPR4207(2); Abcam, USA), LC3B (1:1000; ab192890; Abcam, USA) and β‐Actin (LMAI Bio; Shanghai, China) overnight at 4°C with agitation. The membranes were then warmed to room temperature for 1 h the next day. Next, they were incubated with the secondary antibody (peroxidase‐labelled goat anti‐rabbit IgG; 1:5000; Sigma, USA) for 30 min. The membrane was exposed to an ECL colour‐developing solution (Thermo, Germany), and images were captured with an ECL instrument (Monad, Suzhou, China). Image‐Pro Plus 6.0 software was used to analyse the optical density of the bands. The optical density ratio of the target protein to that of the endogenous protein β‐actin represents the relative content of the target protein and was calculated to compare the differences in protein expression.

### 2.10 Joint intervention with the siRNA and antagomir in Ball‐1 cells for rescue experiments

siRNA targeting EIF4EBP1 was synthesized by Guangzhou Ruibo Biological Company, and Ball‐1 cells were transfected with siEIF4EBP1‐1, siEIF4EBP1‐2 and siEIF4EBP1‐3. After 48 h, EIF4EBP1 expression in the siEIF4EBP1‐1 group was found to be lower than that in the siNC group (for the sequence and screening, see Table S3 and Figure S3). Ball‐1 cells were counted and plated, and siRNA EIF4EBP1 and NC were added. Subsequently, the Lipo2000 transfection reagent was added. After 24 h, half of the sample was separated from the siRNA tube, and miR‐99a‐3p antagomir was added for further culture. After incubation at 37°C for 48 hours, proliferation, cell cycle progression, apoptosis and Western blotting analyses were performed.

### 2.11 miR‐99a‐3p overexpression and interference in an MRL/lpr mouse model

Eighteen MRL/lpr mice (20–30 g, female, 6–8 weeks old, purchased from SPF Biotechnology Co., Ltd.) were divided into groups that received a single high‐dose tail vein injection of miR‐99a‐3p agomir, antagomir or NC at an injection volume of 200 µl; miR‐99a‐3p agomir and antagomir injection doses were 20 nmol and 200 nmol, respectively.^15^, ^16^ Six C57BL/6J (C57) mice (20–25 g, female, 6–8 weeks old) were purchased from Hunan Slack Jingda Experimental Animal Co., Ltd.

Venous blood was collected from the eye, and B lymphocytes were separated using the immunomagnetic bead method, followed by Western blotting and RT‐qPCR analyses. The plasma was retained for ELISA. Mice were euthanized via cervical dislocation, and their kidneys were removed and placed in 4% paraformaldehyde (P0099, Beyotime, China) for fixation. The experimental protocol was approved by the Animal Research Committee of Kunming Medical University (kmmu2021724).

### 2.12 | Coomassie brilliant blue staining method for the measurement of total protein levels in the urine

An appropriate amount of urine was collected and diluted to a standard volume before the measurement of the total protein contents. Equal dilutions of urine in PBS were added to 5 ml of Coomassie brilliant blue solution (Xinfan Biological Technology Co., Shanghai, China). The colour changed from red to blue, and absorbance of the samples was measured at 595 nm.

### 2.13 ELISA for the detection of ANA, dsDNA, IgE, IgM, IL‐6, IL‐10 and BLyS

A total of 100 µl of an HRP‐labelled antibody was added to the wells of the plate provided with the ELISA kit (JL12477‐96T, Jiang Lai Bio, China), followed by incubation at 37°C for 60 min. Next, 50 μl of substrates A and B was added to each well, followed by incubation at 37°C in the dark and the addition of a stop solution. The OD value of each well was measured at a wavelength of 450 nm, and the sample concentration was calculated from the absorbance value based on a standard curve.

### 2.14 HE staining

Longitudinally sectioned kidney tissues from each group were fixed with paraformaldehyde for 24 h and then dehydrated using an ascending gradient of ethanol solutions. The tissues were rendered transparent, embedded in wax, cut into slices with a thickness of 4 µm and incubated at 64°C for 30 min. After dewaxing with xylene, the sections were gradually hydrated using a descending gradient of ethanol solutions and counterstained with haematoxylin (C0105, Beyotime, China) for 4 min. The colour was returned to blue by rinsing with tap water for 20 min before the sections were stained with eosin (C0109, Beyotime, China) for 10 sec. Finally, the sections were incubated with ethanol for dehydration, dewaxed with xylene and mounted with neutral gum. Images were captured using a microscope (Lab. A1, ZEISS, Germany), and the staining results were analysed.

### 2.15 Immunohistochemical (IHC) staining

Tissue slices were prepared, baked and dewaxed. After hydration using a decreasing gradient of alcohol solutions, 0.01 M citric acid buffer was added, and the slices were boiled for 15 min for antigen retrieval. Then, the sections were blocked with 5% BSA (LMAI Bio, Shanghai, China) at room temperature for 30 min and incubated with primary antibodies against EIF4EBP1 (1:100; GTX133182; GeneTex, USA), LAMP‐2A (1:100; EPR4207(2); Abcam, USA) or LC3B (1:100; ab192890; Abcam, USA), which were added in a dropwise manner, overnight at 4°C. The sections were then incubated with the enhancement solution, and the secondary antibody (sheep anti‐mouse; A‐11001; Thermo, Germany) was added in a dropwise manner, followed by incubation at 37°C for 30 min. Next, DAB (ZLI‐9019; Zhongshan Golden Bridge, China) dye solution was added in a dropwise manner, followed by incubation for 5 min. The sections were stained with haematoxylin for 4 min and rinsed with tap water for 20 min to return the colour to blue. The sections were then dehydrated, rendered transparent and mounted. Images were captured, and the positive areas were scanned and calculated. First, a score was assigned according to the staining intensity: 0, colourless; 1, light yellow; 2, brown; and 3, dark brown. The staining intensity was compared with the background colour. Then, a score was assigned according to the percentage of positive cells: 0, negative staining; 1, ≤25% positive cells; 2, 25%–50% positive cells; and 3, >50% positive cells.

### 2.16 Immunofluorescence staining of cells and tissues

Tissue slices were prepared, baked, dewaxed and incubated with a repair solution after hydration with a decreasing gradient of alcohol solutions. An oil pen was used to draw circles around the sections on the slide, and the sections were incubated with diluted serum for blocking. Then, the sections were incubated with an anti‐C3 antibody (1:100; ab11887; Abcam, USA) overnight and then with a secondary goat anti‐rabbit IgG H&L antibody (AmyJet Scientific; Wuhan, China). DIPA (Weifang Bincheng Chemical Industry, China) dyeing solution was then added in a dropwise manner. The samples were placed under a fluorescence microscope (Mshot; Guangzhou, China). Images of 5 fields of view in the centre and surrounding areas of each section were captured, and the positive staining rate was calculated.

The cells were collected and fixed with 4% paraformaldehyde. After blocking, a 1:200 dilution of the primary antibody against LC3B (1:100; ab192890; Abcam, USA) was added to the cells, followed by incubation overnight. Then, a secondary antibody (sheep anti‐mouse; A‐11001; Thermo, Germany) was added, followed by incubation and the addition of DAPI (ID0080; Solarbio, China). The cells were mounted, images were captured, and the positive staining rate was calculated.

### 2.17 Statistical methods

The data are presented as the means±SDs and were analysed using ANOVA and an LSD t test using the SPSS 23.0 software. A two‐tailed *P* value<0.05 was considered significant. The count data were analysed using a chi‐square test. GraphPad Prism 6.0 software was used for the statistical analyses of histograms.

## 3 RESULTS

### 3.1 Verification of differential miR‐99a‐3p expression in patients with SLE

Venous blood from the Tibetan subjects was collected, and the RNA was extracted and subjected to second‐generation high‐throughput sequencing. RT‐qPCR was used to verify the results. The results showed a significant reduction in miR‐99a‐3p expression in Tibetan patients with SLE (Figure 1A, 1B).

RT‐qPCR was used to detect miR‐99a‐3p expression in PBMCs from 10 Tibetan and Han patients with SLE and 10 healthy Tibetan and Han people. miR‐99a‐3p expression in both Tibetan and Han patients with SLE was significantly lower than that in the healthy controls (Figure 1C, 1D). As samples from Tibetan individuals are very difficult to obtain, samples from Han subjects were used in the subsequent experiments.

### 3.2 Functional differences in miR‐99a‐3p expression in Ball‐1, Jurkat, THP‐1 and K562 cells

**FIGURE 2:**
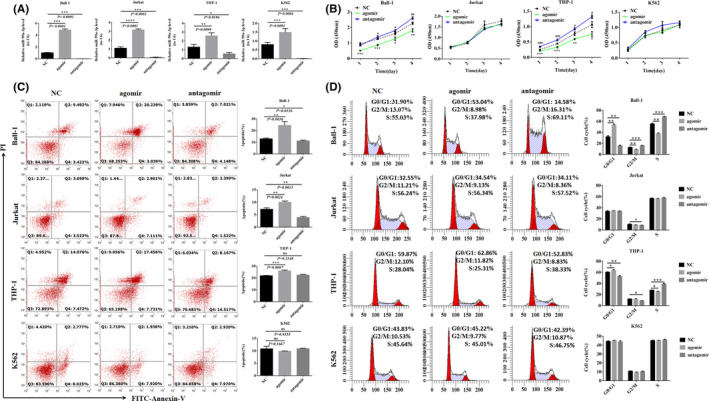
The functional difference in miR‐99a‐3p in Ball‐1, Jurkat, THP‐1 and K562 cells. (A) miR‐99a‐3p expression after transfection of different cells with miR‐99a‐3p agomir, antagomir or NC (*n* = 3). (B) CCK‐8 assays to detect differences in cell proliferation 1, 2, 3 and 4 days after transfection of different cells with miR‐99a‐3p agomir, antagomir or NC (*n* = 4). (C) After transfection of different cells with miR‐99a‐3p agomir, antagomir or NC, flow cytometry was employed to analyse changes in apoptosis in each cell group, and a histogram was constructed to analyse differences in the proportion of apoptosis in each group (Q1, necrosis; Q2, late apoptosis; Q3, normal; and Q4, early apoptosis) (*n* = 3). (D) Different cells were transfected with miR‐99a‐3p agomir, antagomir or NC, and cell cycle changes in each cell group were analysed using flow cytometry; the histogram compares differences in the ratios of cells (*n* = 3) (Note in Fig B: ^*^p < 0.05, ^**^p < 0.01, ^***^p < 0.001, comparison between agomir and NC; ^#^ *p* < 0.05, ^##^ *p* < 0.01, ^###^ *p* < 0.001, antagomir and NC comparison; in Figure D: ^*^p < 0.05, ^**^p < 0.01, ^***^p < 0.001)

Ball‐1, Jurkat, THP‐1 and K562 cells were transfected with the synthesized miR‐99a‐3p agomir and antagomir (see Figure S1), and miR‐99a‐3p expression was detected using RT‐qPCR at 48 h after transfection. Compared with the NC group, miR‐99a‐3p expression increased after miR‐99a‐3p agomir transfection in the four cell lines and decreased after miR‐99a‐3p antagomir transfection in the four cell lines (Figure 2A).

Compared with the NC group, the proliferation of Ball‐1 and THP‐1 cells was reduced on days 1, 2, 3 and 4 after transfection with the miR‐99a‐3p agomir. The opposite effect was observed in Ball‐1 and THP‐1 cells transfected with the miR‐99a‐3p antagomir. However, compared with the NC group, the proliferation of Jurkat and K562 cells was not significantly altered after transfection with the agomir and antagomir (Figure 2B).

Compared with the NC group, the rate of apoptosis of Ball‐1, Jurkat and THP‐1 cells transfected with the miR‐99a‐3p agomir increased significantly. In Ball‐1 and Jurkat cells transfected with the miR‐99a‐3p antagomir, the apoptosis rate was significantly decreased. The apoptosis rates of K562 cells transfected with the agomir and antagomir did not change significantly compared to those of the NC group (Figure 2C).

Compared with the NC group, a higher number of Ball‐1 and THP‐1 cells transfected with the miR‐99a‐3p agomir were observed to be in the G0/G1 phase. The opposite effect was observed in case of Ball‐1 and THP‐1 cells transfected with the miR‐99a‐3p antagomir, that is a lower number of Ball‐1 and THP‐1 cells transfected with the miR‐99a‐3p antagomir were observed to be in the G0/G1 phase, compared with the case in the NC group.

Compared with the NC group, the number of Ball‐1 cells transfected with the miR‐99a‐3p agomir that were in the G2/M and S phases was reduced. The opposite effect was observed in case of Ball‐1 cells transfected with the miR‐99a‐3p antagomir. In Jurkat cells transfected with the miR‐99a‐3p antagomir, the number of cells in the G2/M phase was lower than that in the NC group (*p* < 0.05). The proportion of K562 cells transfected with the agomir and antagomir in all phases of the cell cycle did not demonstrate any significant changes compared with the case for the cells from the NC group (Figure 2D). Changes observed in the proliferation, apoptosis and cell cycle distribution of Ball‐1 cells after transfection with the miR‐99a‐3p agomir, antagomir and NC were relatively stable.

### 3.3 Confirmation of the target genes

The target genes of miR‐99a‐3p were predicted using TarBase (http://mirtarbase.mbc.nctu. edu.tw/php/index.php), miRDB (http://www.mirdb.org), TargetScan, (http://www.targetscan.org/vert_71) and miRBase (http://www.mirbase.org). Based on a literature search, we selected EIF4EBP1, NCAPG, IKBKB and PRKCB for further analysis.

**FIGURE 3:**
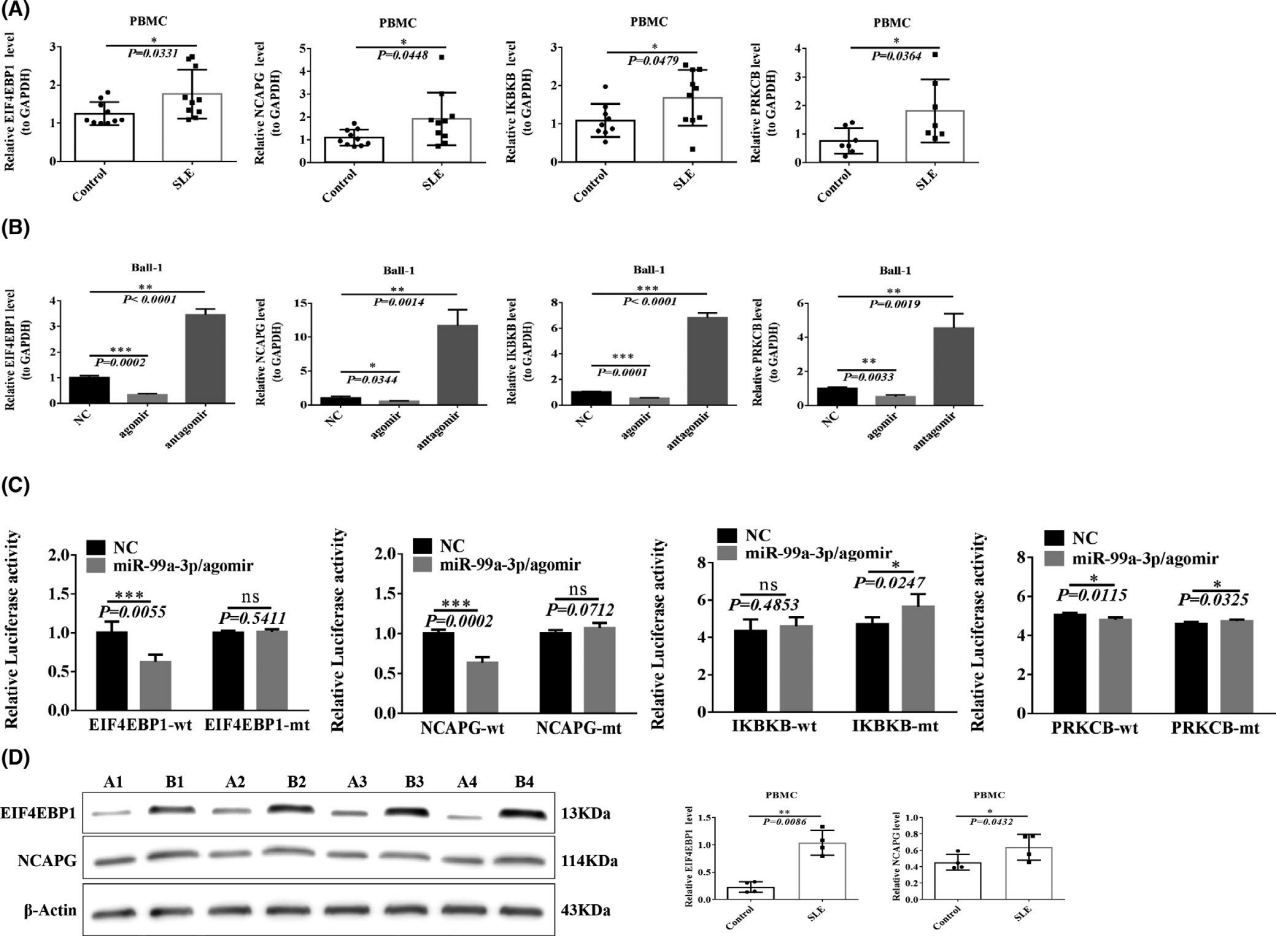
Target gene confirmation. (A) RT‐qPCR was used to detect the predicted target gene expression in SLE patients and healthy controls (*n* = 10). (B) Changes in target genes after transfection of Ball‐1 cells with miR‐99a‐3p agomir, antagomir or NC (*n* = 3). (C) Transfection of 293T cells with mutant or wild‐type fluorescein vectors carrying binding sites and agomir, the fluorescein report value was read with a fluorescence spectrophotometer, and the difference in the influence of agomir between mutant and wild‐type was analysed (n = 5). (D) Western blotting verified the differences in EIF4EBP1 and NCAPG expression in SLE patients (B1, B2, B3, B4) and healthy people (A1, A2, A3, A4) (*n* = 3)

RT‐qPCR was performed to detect the expression of EIF4EBP1, NCAPG, IKBKB and PRKCB in PBMCs isolated from Han patients with SLE and healthy Han individuals. The expression levels of these genes were significantly upregulated in the PBMCs derived from patients with SLE (Figure 3A).

Ball‐1 cells were transfected with the miR‐99a‐3p agomir, antagomir and NC, and target gene expression was detected using RT‐qPCR 48 h after transfection. Compared with the NC group, the expression of all target genes decreased in Ball‐1 cells transfected with the miR‐99a‐3p agomir, but increased in Ball‐1 cells transfected with the miR‐99a‐3p antagomir (Figure 3B).

293T cells were transfected with hsa‐miR‐99a‐3p+EIF4EBP1‐WT. The fluorescence intensity in the miR‐99a‐3p/agomir+EIF4EBP1‐WT group was significantly reduced (*p* = 0.0055) compared with that in the NC+EIF4EBP1‐WT group. Meanwhile, 293T cells were transfected with hsa‐miR‐99a‐3p+EIF4EBP1‐MT, and their fluorescence intensity was compared with that of the NC+EIF4EBP1‐MT group; the fluorescence intensity did not change significantly after the transfection (*p* = 0.5411). The predicted position revealed a targeting relationship between hsa‐miR‐99a‐3p and EIF4EBP1 (see Figure S2).

The fluorescence intensity in the miR‐99a‐3p/agomir+NCAPG‐WT group was significantly reduced (*p* = 0.0002) compared with that in the NC+NCAPG‐WT group. 293T cells were transfected with hsa‐miR‐99a‐3p+NCAPG‐MT, and their fluorescence intensity was compared with that of the NC+NCAPG‐MT group. The fluorescence intensity of 293T cells did not change significantly after the transfection (*p* = 0.0712), and the predicted position revealed a targeting relationship between hsa‐miR‐99a‐3p and NCAPG. hsa‐miR‐99a‐3p did not demonstrate any targeting relationship with IKBKB or PRKCB (Figure 3C).

Western blotting further verified the expression of target genes in patients with SLE and healthy controls. The EIF4EBP1 and NCAPG levels were significantly increased in patients with SLE (Figure 3D).

### 3.4 miR‐99a‐3p participates in the autophagy signalling pathway by exerting effects on target genes

**FIGURE 4:**
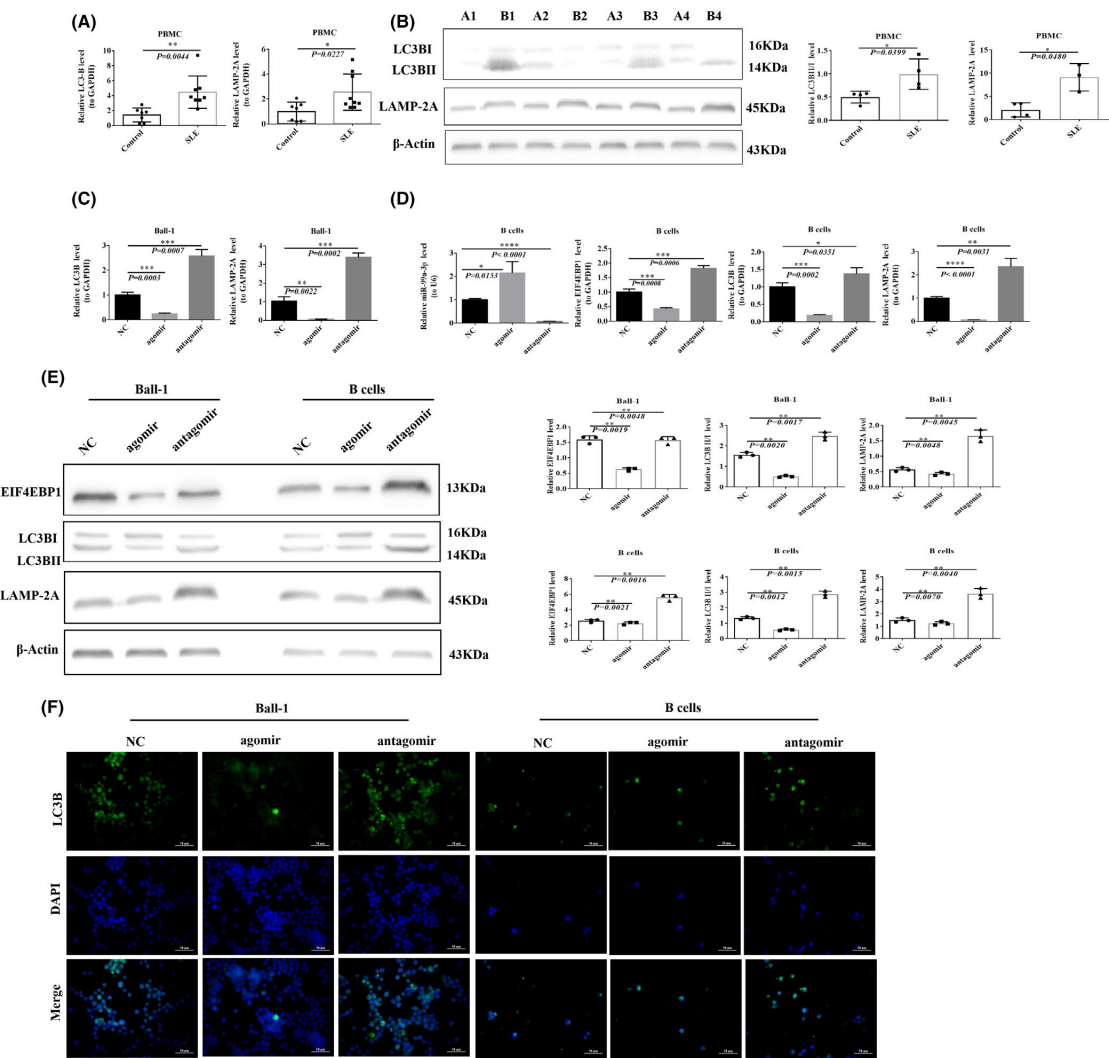
miR‐99a‐3p participates in the autophagy signalling pathway through target genes. (A) RT‐qPCR detection of LC3B and LAMP‐2A expression in PBMCs from SLE patients and healthy controls (*n* = 10). B) Western blotting detection of LC3B and LAMP‐2A expression in SLE patients (B1, B2, B3, B4) and healthy controls (A1, A2, A3, A4) (*n* = 3). (C) RT‐qPCR detection of LC3B and LAMP‐2A expression after transfection of Ball‐1 cells with miR‐99a‐3p agomir, antagomir or NC (*n* = 3). (D) RT‐qPCR detection of miR‐99a‐3p, EIF4EBP1, LC3B and LAMP‐2A expression after transfection of B cells with miR‐99a‐3p agomir, antagomir or NC. (E) Western blotting detection of EIF4EBP1, LC3B and LAMP‐2A protein expression after transfection of Ball‐1 and B cells with miR‐99a‐3p agomir, antagomir or NC (*n* = 3). (F) IF of LC3B after transfection of Ball‐1 and B cells with miR‐99a‐3p agomir, antagomir or NC (×400; scale bar, 50 µm). Green fluorescence indicates positive staining for LC3B, blue fluorescence represents nuclear staining with DAPI, and merged fluorescence images show both LC3B and DAPI staining (*n* = 3)

RT‐qPCR was used to detect LC3B and LAMP‐2A expression in PBMCs isolated from patients with SLE and healthy controls. Higher LC3B and LAMP‐2A expression levels were observed in patients with SLE than those in the healthy control group (Figure 4A). Western blotting was used to analyse the levels of the autophagy pathway marker proteins LC3B and LAMP‐2A in PBMCs isolated from patients with SLE and healthy controls. The ratio of the levels of these two mRNAs was higher in patients with SLE (*p* = 0.0399, Figure 4B).

Ball‐1 cells were transfected, and the expression levels of LC3B and LAMP‐2A were detected using RT‐qPCR 48 h after transfection. Transfection with the miR‐99a‐3p agomir decreased the levels of both LC3B and LAMP‐2A in Ball‐1 cells, but transfection with the miR‐99a‐3p antagomir increased the levels of these genes (Figure 4C).

Compared with the NC group, miR‐99a‐3p expression increased after the transfection of B cells with the miR‐99a‐3p agomir (*p* = 0.0153), whilst the levels of EIF4EBP1, LC3B and LAMP‐2A decreased. After the transfection of B cells with the miR‐99a‐3p antagomir, miR‐99a‐3p expression decreased (*p* < 0.0001), whilst the levels of EIF4EBP1, LC3B and LAMP‐2A increased (Figure 4D).

A total of 48 h after the transfection of Ball‐1 and B cells, the protein levels of EIF4EBP1, LC3B and LAMP‐2A decreased significantly in the miR‐99a‐3p agomir group, but increased significantly in the miR‐99a‐3p antagomir group, compared to those in the NC group (Figure 4E).

The intensity of IF staining for LC3B expression in Ball‐1 and B cells was weaker in the miR‐99a‐3p agomir group than in the NC group but stronger in the miR‐99a‐3p antagomir group than in the NC group, as observed by fluorescence microscopy (Figure 4F).

### 3.5 Changes in the functions of Ball‐1 cells after a rescue experiment

**FIGURE 5:**
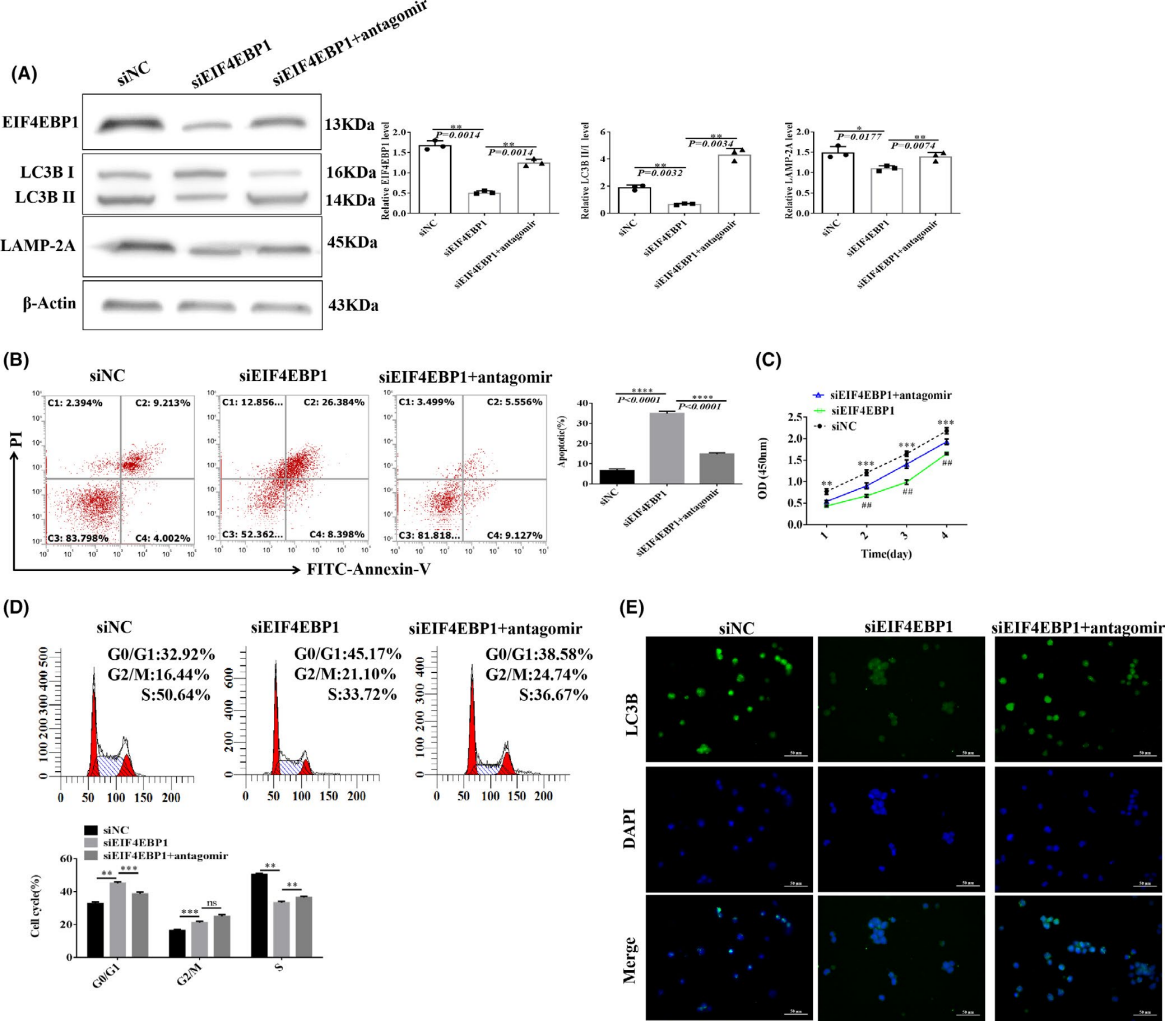
Ball‐1 cell function changes after a rescue experiment. (A) Western blotting was used to detect EIF4EBP1, LC3B, and LAMP‐2A expression after transfection of Ball‐1 cells with siNC, siEIF4EBP1 or siEIF4EBP1+antagomir (*n* = 3). (B) After transfection of Ball‐1 cells with siNC, siEIF4EBP1, or siEIF4EBP1+antagomir, flow cytometry was used to analyse changes in apoptosis in each cell group, and a histogram was constructed to analyse differences in the proportion of apoptosis in each group (C1, necrosis; C2, late apoptosis; C3, normal; and C4, early apoptosis) (*n* = 3). (C) CCK‐8 assays to detect differences in the proliferation of Ball‐1 cells after transfection with siNC, siEIF4EBP1 or siEIF4EBP1+antagomir at 1, 2, 3 and 4 days (*n* = 3). (D) After transfection of Ball‐1 cells with siNC, siEIF4EBP1 or siEIF4EBP1+antagomir, flow cytometry was used to analyse cell cycle changes in each cell group; the histogram compares differences in the proportion of cells in each group (*n* = 3). (E) IF of LC3B after transfection of Ball‐1 cells with siNC, siEIF4EBP1 or siEIF4EBP1+antagomir (×400; scale bar, 50 µm). Green fluorescence indicates positive staining for LC3B, blue fluorescence represents nuclear staining with DAPI, and merged fluorescence images show both LC3B and DAPI staining (n = 3) (Note in Figure C: ^**^p < 0.01, ^***^p < 0.001, comparison between siEIF4EBP1 and siNC; ^##^ *p* < 0.01, comparison between siEIF4EBP1+antagomir and siEIF4EBP1; Figure D: ^**^p < 0.01, ^***^p < 0.001; ns, not significant

Western blotting was used to detect the levels of EIF4EBP1, LC3B and LAMP‐2A in Ball‐1 cells transfected with siNC, siEIF4EBP1 or siEIF4EBP1+antagomir. Lower EIF4EBP1, LC3B and LAMP‐2A protein levels were detected in Ball‐1 cells transfected with siEIF4EBP1 than those in the siNC group. The EIF4EBP1, LC3B and LAMP‐2A proteins were expressed at significantly higher levels in Ball‐1 cells transfected with siEIF4EBP1+antagomir than in those transfected with siEIF4EBP1 (Figure 5A).

After Ball‐1 cells were transfected with siEIF4EBP1, the apoptotic rate, as determined by flow cytometry, was significantly higher than that in the siNC group, and the apoptosis rate was significantly lower in cells transfected with siEIF4EBP1+antagomir than that in cells transfected with siEIF4EBP1 (Figure 5B).

The proliferation of transfected Ball‐1 cells in the siEIF4EBP1 group was lower when observed on days 1, 2, 3 and 4, than that of cells in the siNC group. The proliferation of cells in the siEIF4EBP1+antagomir group was higher when observed on days 2, 3 and 4, than that of cells in the siEIF4EBP1 group (Figure 5C).

After transfection with siEIF4EBP1, higher numbers of cells were observed in the G0/G1 and G2/M phases than those in the siNC group, and the number of cells in the S phase was lower than that in the siNC group. After transfection with siEIF4EBP1+antagomir, the number of cells in the G0/G1 phase decreased, and the number of cells in the G2/M and S phases increased compared to that in the siEIF4EBP1 group (Figure 5D). Fluorescence microscopy showed that the LC3B IF staining intensity in the siEIF4EBP1 group was lower than that in the siNC group, and the LC3B IF staining intensity was higher in the siEIF4EBP1+antagomir group than that in the siEIF4EBP1 group (Figure 5E).

### 3.6 Disease progression in the mouse MRL/lpr model after experimental intervention with miR‐99a‐3p under *in vivo* conditions

**FIGURE 6:**
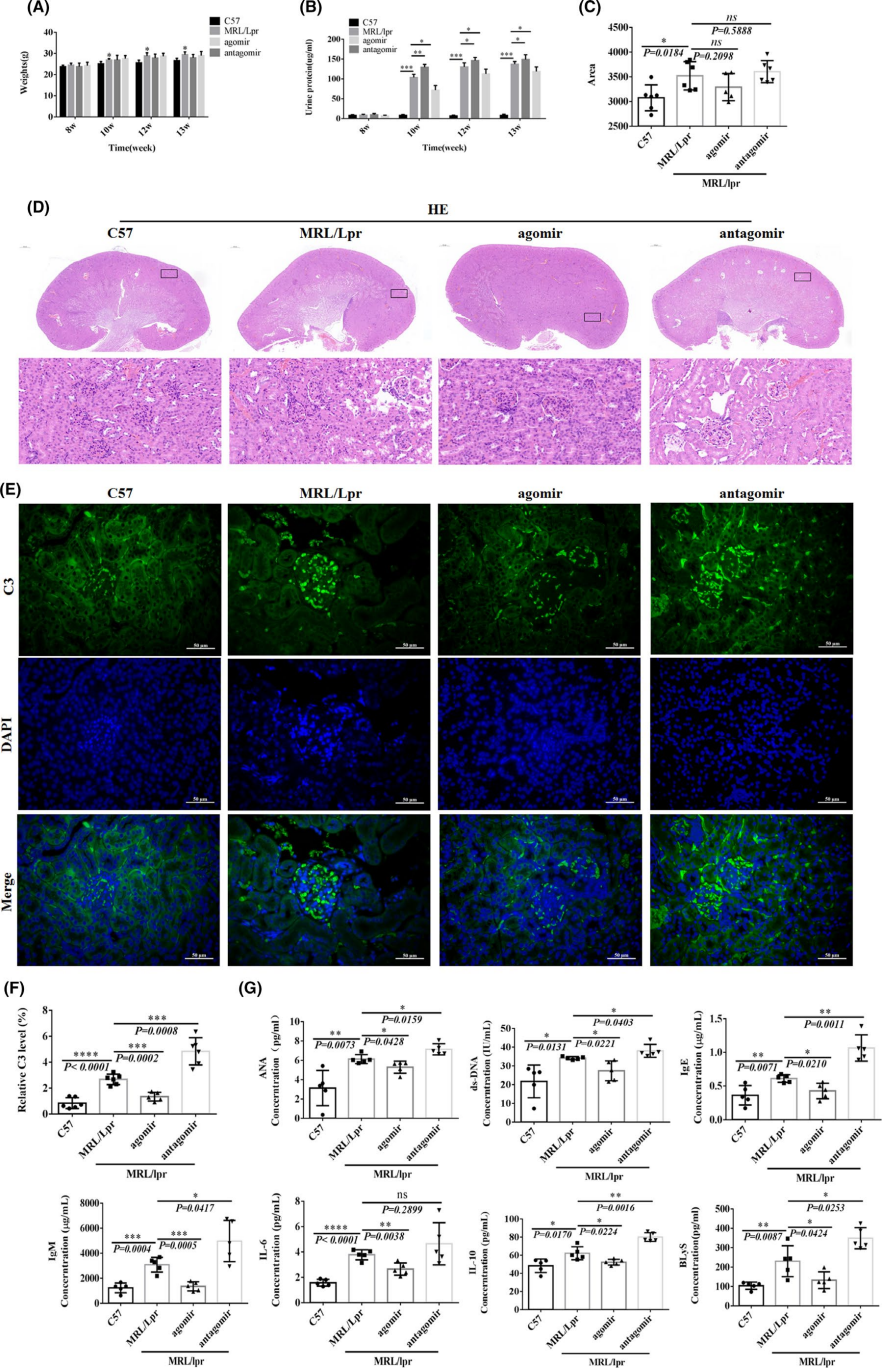
Disease progression in MRL/lpr lupus mice after experimental intervention with miR‐99a‐3p in vivo. (A) Bodyweight changes in MRL/lpr and C57 mice 8 w, 10 w, 12 w and 13 w after intervention (*n* = 6). (B) Coomassie brilliant blue staining to detect changes in urine protein in MRL/lpr and C57 mice 8 w, 10 w, 12 w and 13 w after intervention (*n* = 3). (C) HE staining of glomerular area in MRL/lpr and C57 mice after intervention (*n* = 3). (D) HE staining of MRL/lpr and C57 mice after intervention (×2.5 and ×400). (E) C3 IF in MRL/lpr and C57 mice after intervention (×400; scale bar, 50 µm). Green fluorescence indicates positive C3 staining, blue fluorescence represents DAPI nuclear staining, and merged fluorescence images show C3 and DAPI staining. F) C3 IF in MRL/lpr and C57 mice after intervention (*n* = 3). (G) ELISA detection of ANA, dsDNA, IgE, IgM, IL‐6, IL‐10 and BLyS expression in MRL/lpr and C57 mice after intervention (*n* = 6) (^*^ *p* < 0.05, ^**^ *p* < 0.01, ^***^ *p* < 0.001; ns, not significant)

The bodyweight of the mice in the four groups increased as the number of feeding weeks increased. One mouse in the agomir group died at 12 w, and the bodyweight of mice in the MRL/Lpr group was higher than that of mice in the C57 group at 10 w, 12 w and 13 w. However, the miR‐99a‐3p intervention did not significantly alter the bodyweight (Figure 6A). At 13 w, the hair around the nose and eyes of mice in the MRL/lpr group was shed, and the animals exhibited fewer and slower behavioural responses. The mice from the antagomir group presented alopecia areata around the nose, eyes and forehead, and their behavioural responses were slower.

No difference in the urine protein levels was observed between the four groups at 8 weeks (*p* > 0.05). However, at 10 w, 12 w and 13 w, the urine protein levels increased significantly in mice from all the groups, except the C57 group. The urine protein levels of mice in the MRL/Lpr and antagomir groups were higher than those of mice in the C57 group, and the urine protein levels in the mice from the agomir group were lower than those in the mice from the C57 group (Figure 6B).

CaseViewer 3.3 was used to measure the glomerular area. The glomerular area in the MRL/lpr group was larger than that in the C57 group (*p* = 0.0184), suggesting that the mice in the MRL/lpr group experienced some glomerular oedema. The glomerular area in the agomir group was smaller than that in the MRL/lpr group (*p* = 0.2098), and the glomerular area in the antagomir group was greater than that in the MRL/lpr group (*p* = 0.5888), but these two differences were not significant (Figure 6C, 6D).

IF staining for C3 deposition was assessed in MRL/lpr mice and C57 mice after the intervention, and C3 deposition was higher in the MRL/lpr group than in the C57 group (*p* < 0.0001). C3 deposition in the agomir group was lower than that in the MRL/lpr group (*p* = 0.0002). C3 deposition in the antagomir group was higher than that in the MRL/lpr group (*p* = 0.0008, Figure 6E, 6F).

The levels of ANA, dsDNA, IgE, IgM, IL‐6, IL‐10 and B lymphocyte stimulator (BLyS) in the MRL/lpr group were higher than those in the C57 group, as observed by ELISA. The levels of the same indices in the agomir group were lower than those in the MRL/lpr group. The mice in the antagomir group showed higher levels of all indices, except for IL‐6, than those in the MRL/lpr group (Figure 6G).

### 3.7 Changes in the expression of target genes and proteins associated with the related pathways in the mouse MRL/lpr lupus model after experimental intervention with miR‐99a‐3p under *in vivo* conditions

**FIGURE 7:**
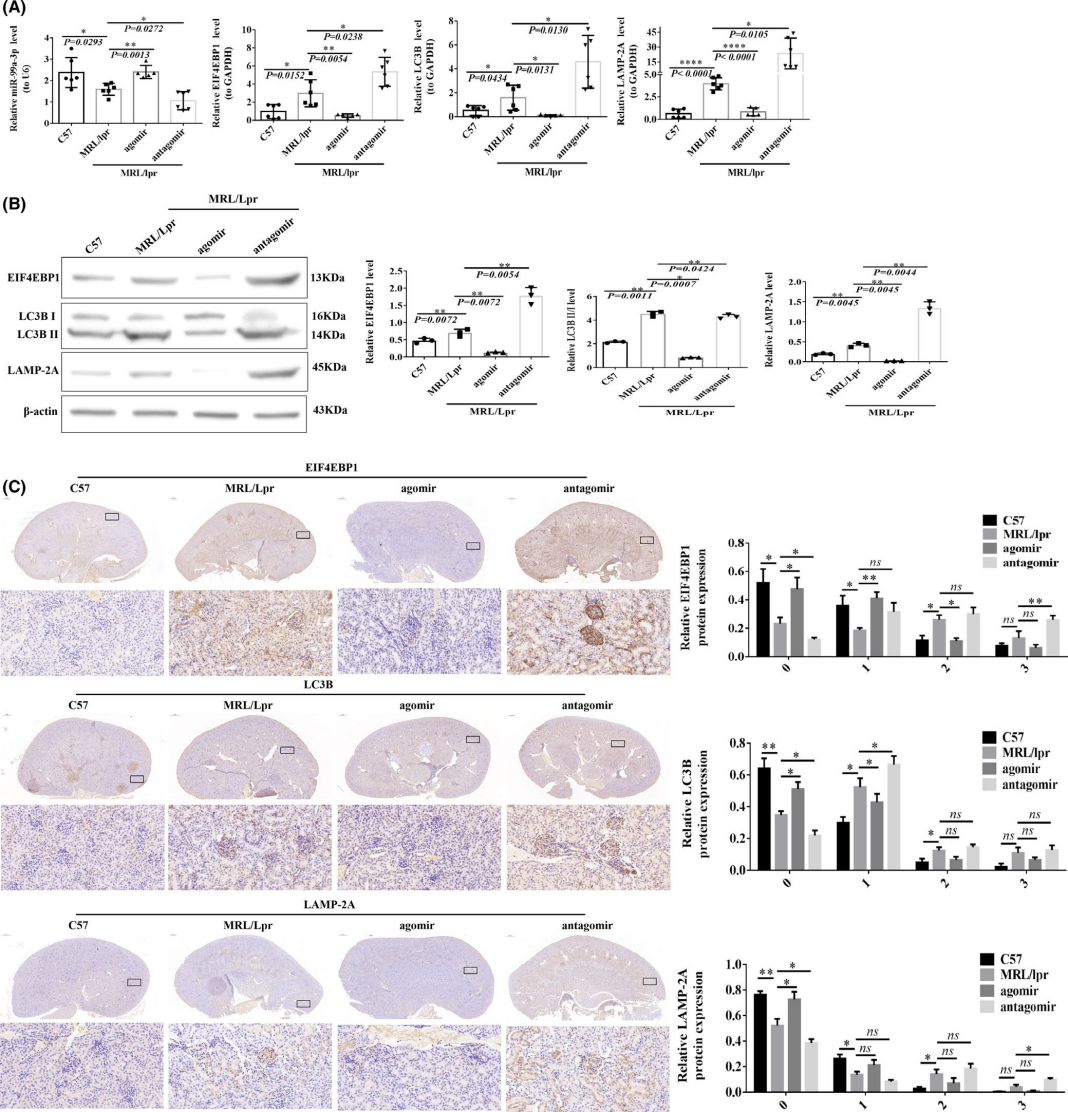
Changes in target genes and pathway proteins in MRL/lpr lupus mice after experimental miR‐99a‐3p intervention in vivo. (A) RT‐qPCR detection of miR‐99a‐3p, EIF4EBP1, LC3B and LAMP‐2A changes after intervention (*n* = 6). (B) Western blotting detection of EIF4EBP1, LC3B and LAMP‐2A protein expression after intervention (*n* = 6). (C) IHC staining of EIF4EBP1, LC3B and LAMP‐2A after intervention (*n* = 6) (×2.5 and ×400; brown‐yellow indicates positive staining; ^*^p < 0.05, ^**^p < 0.01; ns, not significant)

After injection of the appropriate constructs via the tail vein, miR‐99a‐3p expression was observed to be lower in the mice from the MRL/lpr group than in those from the C57 group (*p* = 0.0293). miR‐99a‐3p expression in the mice from the agomir group was higher than that in mice from the MRL/lpr group (*p* = 0.0013); the miR‐99a‐3p level in mice from the antagomir group was lower than that in the mice from the MRL/lpr group (*p* = 0.0272). EIF4EBP1, LC3B and LAMP‐2A were expressed at higher levels in the mice from the MRL/lpr group than in those from the C57 group. The EIF4EBP1, LC3B and LAMP‐2A mRNA expression levels in the mice from the agomir group were lower than that in the mice from the MRL/lpr group. EIF4EBP1, LC3B and LAMP‐2A mRNAs were expressed at significantly higher levels in the mice from the antagomir group than in those from the MRL/lpr group (Figure 7A).

EIF4EBP1, LC3B and LAMP‐2A were expressed at higher levels in the mice from the MRL/lpr group than in those from the C57 group, as determined by the Western blotting analysis. The expression levels of EIF4EBP1, LC3B and LAMP‐2A in mice from the agomir group were lower than those in the mice from the MRL/lpr group. EIF4EBP1, LC3B and LAMP‐2A were expressed at higher levels in the mice from the antagomir group than in those from the MRL/lpr group (Figure 7B).

IHC staining analysis showed the low staining intensities of EIF4EBP1, LC3B and LAMP‐2A in the kidneys of C57 mice, whilst the staining intensities were stronger in the kidneys of mice from the MRL/lpr group. The samples from mice in the antagomir group showed a significant increase in staining intensity, and staining was significantly weakened in the samples from mice in the agomir group (Figure 6C). The EIF4EBP1, LC3B and LAMP‐2A expression levels in the mice from the MRL/lpr group were lower than in those from the C57 and agomir groups with scores of 0 points, and significantly higher than in those from the antagomir group. As the score increased, the intensity of IHC staining and the percentage of positive cells in the C57 and agomir groups gradually decreased, whilst the opposite trend was observed in the antagomir group (Figure 7C).

## 4 DISCUSSION

SLE is a disease of the immune system caused by the overactivation of immune cells and the secretion of large amounts of autoantibodies. Overactive B cells are involved in almost all aspects of the pathogenesis of SLE. Although significant progress has been made in treatments for SLE, treatment is ineffective for some patients. Many patients frequently experience relapse.

miRNAs are endogenous noncoding RNAs that are widely distributed in the human body and play important roles in regulating cell proliferation and apoptosis, and SLE progression. Importantly, miRNA‐based biomarkers and treatment methods may become viable options for the treatment of SLE.^5^

Zhang et al.^17^ elaborated the role of the circRNA–miRNA–mRNA regulatory network in SLE, and 29 DECs (2 upregulated and 27 downregulated) were identified in individuals with SLE. Latini et al.^18^ reported that miR‐155, miR‐499a and miR‐142 are involved in the pathogenesis and determining the clinical phenotype of SLE. Tao et al.^19^ reported that miR‐152‐3p promotes the Toll‐like receptor (TLR)‐mediated CD4+ T cell inflammatory response by regulating the DNMT1/MyD88 signalling pathway, which may be considered as a new target for SLE treatment.

In the present study, miR‐99a‐3p expression was found to be significantly decreased in Tibetan and Han patients with SLE, consistent with the results of a study performed by Pradhan in the Indian population,^12^ the study performed by Jin in the Korean population,^13^ and the study performed by Frangou in the European population.^14^ No previous reports have documented miR‐99a‐3p expression in Chinese patients with SLE. Obtaining samples from Han patients with SLE is more convenient, and thus, the Han nationality was selected as the research subject for subsequent experiments.

We selected the Ball‐1, Jurkat, THP‐1 and K562 cell lines to elucidate and verify the function of miR‐99a‐3p in different immune cells. After Ball‐1 cells were transfected, cell proliferation, apoptosis and cell cycle changes were found to be relatively stable. Here, Ball‐1 cells were selected for further experiments. In the pathogenesis of SLE, B cells not only produce autoantibodies but also regulate the activation of T cells via modulating the levels of various cytokines and antigen presentation processes, which also aggravate the progression of SLE.^20^ Therefore, studies examining how B cells play a role in SLE and produce pathogenic autoantibodies via signal transmission between cells are particularly important.

Using bioinformatics methods, we predicted that EIF4EBP1 was a target gene of miR‐99a‐3p. In the present study, 293T cells were transfected with a luciferase reporter gene vector containing the WT/MT 3'‐UTR of EIF4EBP1. The results confirmed that miR‐99a‐3p interacted with the complementary 3'‐UTR sequence of EIF4EBP1 and downregulated the expression of EIF4EBP1.

EIF4EBP1 is an inhibitor of translation initiation, and its activity is regulated by preventing the assembly of eIF4E into the eIF4F complex. The role of EIF4EBP1 in SLE pathogenesis has not yet been reported.

Autophagy is a process whereby a cell engulfs its own cytoplasmic proteins or organelles in vesicles that then fuse with lysosomes. In patients with SLE, autophagy plays a role in the identification and removal of pathogens, antigen processing and antigen presentation. At the same time, autophagy maintains the stability of the intracellular environment by regulating the levels of cytokines and controlling intracellular energy and metabolism. More autophagosomes were detected in T and B lymphocytes isolated from patients with SLE, and the level of autophagy was significantly increased. If autophagy is abnormal, the presentation and processing of antigens are hindered. Although the role of autophagy in the pathogenesis of SLE has not yet been completely elucidated, autophagy is closely related to the progression of SLE.

Many studies have confirmed that miRNAs regulate autophagy.^21^, ^22^ Abnormal autophagy leads to the accumulation of apoptotic cells and induces the production of autoantibodies. EIF4EBP1 regulates the activity of mTORC1, thereby inducing autophagy,^23^ and LC3 is a key protein involved in autophagy.^24^ LAMP‐2A is a key regulatory protein in the chaperone‐mediated autophagy (CMA) pathway. Inhibition of the expression of the LAMP‐2A protein specifically blocks the CMA pathway.^25^

This study further confirmed that transfection with the miR‐99a‐3p agomir reduced the expression of the autophagy‐related genes LC3B and LAMP‐2A in clinical samples and cells (Ball‐1 and B cells) under *in vitro* and *in vivo* (MRL/lpr mouse B cells) conditions. Transfection with miR‐99a‐3p antagomir exerted the opposite effect, suggesting that miR‐99a‐3p negatively regulates autophagy. The trend observed in the changes in EIF4EBP1 expression was consistent with the trend in the changes in the autophagy level, suggesting that EIF4EBP1 exerts a positive regulatory effect on autophagy.

In the present study, a rescue experiment was conducted to confirm the mode of interaction between miR‐99a‐3p and EIF4EBP1. Notably, miR‐99a‐3p affected cell proliferation and apoptosis by modulating the expression of target genes.

BLyS is a newly identified member of the tumour necrosis factor family and is involved in the regulation of B cell proliferation and antibody production. Transgenic mice showing the overexpression of BLyS demonstrate an increased number of B cells, increased serum ANA and dsDNA levels, and immunoglobulin deposition in the kidney.^26^ Benlysta was the first inhibitor designed to target BLyS; it binds to soluble BLyS with high affinity and inhibits its activity to achieve disease control.^27^

In this study, an *in vivo* experimental model was constructed using MRL/Lpr mice by injecting a miR‐99a‐3p agomir or antagomir or a NC into the tail vein of the mice. The levels of ANA, dsDNA, IgE, IgM, IL‐6, IL‐10 and BLyS in mice from the antagomir group increased significantly, and the SLE disease activity was stronger. In the antagomir group, urinary protein levels and C3 IF deposition in the kidneys were increased, and kidney damage was more serious, which is consistent with results from a previous report.^28^ These results indicate that miR‐99a‐3p inhibition exacerbated SLE progression. However, the IgM levels were higher in mice from the antagomir group than in the mice from the MRL/lpr group. This observation contradicts those of a previous study.^29^ This finding may be related to the euthanization of 13‐week‐old mice in this study; the animals may still have been in the early stages of negative feedback.

## 5 CONCLUSIONS

This study is the first to report that miR‐99a‐3p, which targets EIF4EBP1, participates in the autophagy signalling pathway and affects the function of B cells, thereby aggravating SLE progression. This study provides a deeper understanding of the molecular mechanisms underlying the activity of miRNA regulatory networks associated with SLE pathogenesis. The abnormal expression patterns of miR‐99a‐3p and its target gene EIF4EBP1 observed in the present study are expected to be considered potential therapeutic targets in patients with SLE.

## 6 LIMITATIONS

This study had a few limitations. First, most of the experiments in this study were performed using Ball‐1 and B cells, but some differences were observed between Ball‐1 and B cells derived from healthy controls and B cells obtained from patients with SLE. The results obtained from the B cells used in this study were not ideal. Second, the rescue experiment did not use gene knockout mice, and thus, verification of the results of the rescue experiment by means of relevant functional experiments is required. Third, due to various restrictions, Tibetan patients were not directly involved in further studies. The number of clinical cases was small, and correlation analysis between clinical indicators and laboratory indicators was not performed. Fourth, the relationship between miR‐99a‐3p and NCAPG and their roles in the pathogenesis of SLE were not studied.

## CONFLICT OF INTEREST

The authors have no proprietary interest in any aspect of the study.

## AUTHOR CONTRIBUTIONS

**Meng Yang:** Formal analysis (equal); Methodology (lead); Software (equal); Writing‐original draft (lead). **Binbin Yang:** Formal analysis (equal); Software (equal); Validation (lead). **Danqi Deng:** Conceptualization (lead); Funding acquisition (lead); Supervision (lead).

## Supporting information

Supplementary Material

## Data Availability

All data sets generated for this study are included in the article/Supplementary Material.
