# Supplementary material for: Targeting of EIF4EBP1 by miR‐99a‐3p affects the functions of B lymphocytes via autophagy and aggravates SLE disease progression
Source: J Cell Mol Med. 2021 Oct 20;25(21):10291–305. doi: 10.1111/jcmm.16991 (PMC8572797; doi:10.1111/jcmm.16991)
Supplement: Supplementary file 1 — Supplementary Material [file JCMM-25-10291-s001.docx]

| Table 1. General clinical data of the study subjects (x̅±s) | | | | | |
| --- | --- | --- | --- | --- | --- |
| Group | Tibetan patients with SLE | Healthy Tibetan controls | Han patients with SLE | Healthy Han controls | *P* |
| Number | n=10 | n=10 | n=10 | n=10 |  |
| Age | 46.1±16.4 | 39.7 ±3.76 | 38.2±5.5 | 36.8 ±5.3 | 0.1346 |
|  |  |  |  |  |  |
| Female/Male | 10/0 | 10/0 | 10/0 | 10/0 | 1 |
| Disease duration | 7.8±2.62 | - | 7.5±3.15 | - | 0.8195 |
|  |  |  |  |  |  |
| [Altitude](javascript:;) | 3171.6±222.64 | 3079 ±74.35 | 1605±105.26 | 1601 ±85.57 | < 0.0001 |
| ANA, n (%) | 9(90%) | - | 10(100%) | - | 1 |
| Anti-ds-DNA, n (%) | 3(30%) | - | 4(40%) | - | 1.0000 |
|  |  |  |  |  |  |
| Anti-Sm, n (%) | 3(30%) | - | 3(30%) | - | 1 |
| Anti-Ro, n (%) | 4(40%) | - | 3(30%) | - | 1 |
| Anti-SSA, n (%) | 3(30%) | - | 2(20%) | - | 1 |
| Anti-U1RNP, n (%) | 4(40%) | - | 4(40%) | - | 1 |
| Corticosteroid therapy, n (%) | 10(100%) | - | 10(100%) | - | 1 |
| Immunosuppressive therapy n (%) | 6(60%) | - | 7(70%) | - | 1 |
| Disease activity (SLEDAI, mean ± SD) | 7.400±3.03 | - | 8.567±3.82 | - | 0.4589 |

Table 2. Sequence of primers for detection

| Primer name | Primer sequence (5'-3') | |
| --- | --- | --- |
| hsa-miR-99a-3p-F | CCCATTGGCATAAACCCGTA | |
| hsa-EIF4EBP1-F | GGGTCACCAGCCCTTCC |  |
| hsa-EIF4EBP1-R | CCCGCCCGCTTATCTTC |  |
| hsa-NCAPG-F | TCAAATCCAGAAGTTAGACGGG |  |
| hsa-NCAPG-R | TTCAAATATTCACAAAGGGCAC |  |
| hsa-IKBKB-F | TTCGCTACCCTTCCCCAATA |  |
| hsa-IKBKB-R | AGCCACTTCTCCAGCCGTTC |  |
| hsa-PRKCB-F | GAGCAAACAGAAGACCAAAACC |  |
| hsa-PRKCB-R | GTGGCACAGGCACATTGAAGTA |  |
| hsa-LC3-Ⅱ-F | CGAACAAAGAGTAGAAGATGTCCGA |  |
| hsa-LC3-Ⅱ-R | GCCTGATTAGCATTGAGCTGTAAGC |  |
| hsa-LAMP-2A-F | TCCAAAGGAAAAACCAGAAGC |  |
| hsa-LAMP-2A-R | TAGAGCAGTGTGAGAACGGCA |  |
| hsa-GAPDH-F | CGCTGAGTACGTCGTGGAGTC |  |
| hsa-GAPDH-R | GCTGATGATCTTGAGGCTGTTGTC |  |
| hsa-U6 | GCAGCACATATACTAAAATTGGAAC |  |
| mmu-miR-99a-3p-F | CAAGCTCGTTTCTATGGGTCT |  |
| mmu-EIF4EBP1-F | TTTCTGATGGAGTGTCGG |  |
| mmu-EIF4EBP1-R | TCCTTTGGGGGTGTTTTG |  |
| mmu-LC3-Ⅱ-F | CTAACCAAGCCTTCTTCCTCCT |  |
| mmu-LC3-Ⅱ-R | GCCGTCTTCATCTCTCTCACTC |  |
| mmu-LAMP-2A-F | ATTTGGATTCGCTGTCTCTTGG |  |
| mmu-LAMP-2A-R | GTGGTGGGAGTTTGGTCTTCTT |  |
| GAPDH-F | CCTTCCGTGTTCCTACCCC |  |
| GAPDH-R | GCCCAAGATGCCCTTCAGT |  |
| U6 | CTCGCTTCGGCAGCACA |  |

| Table 3. genOFFTM st-h-EIF4EBP1 sequence report | | |
| --- | --- | --- |
| Product number | Product name | Target sequence |
| stB0006228A | genOFFTM st-h-EIF4EBP1_001 | GTACCAGGATCATCTATGA |
| stB0006228B | genOFFTM st-h-EIF4EBP1_002 | GCAATAGCCCAGAAGATAA |
| stB0006228C | genOFFTM st-h-EIF4EBP1_003 | GATGGAGTGTCGGAACTCA |
| stB0006228D | siNC | ACAGAAGCGATTGTTGATC |

Supplementary figures
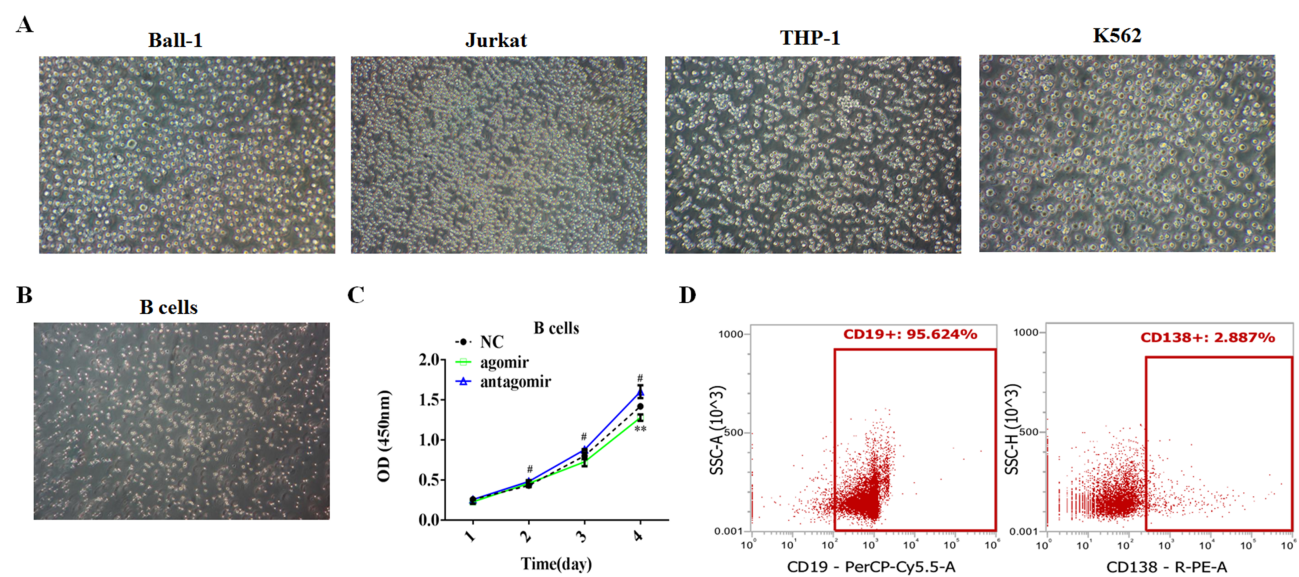
 1. Cell culture, identification and proliferation of B cells. (A) Ball-1, Jurkat, THP-1, and K562 cell appearance under a microscope (×200). (B) Appearance of B cells under a microscope (×200). (C) CCK-8 assays to detect the proliferation of B cells 1, 2, 3, and 4 days after transfection with miR-99a-3p agomir, antagomir, or NC (*^**^P*<0.01*,* comparison between agomir and NC; ^#^*P*<0.05, comparison between antagomir and NC). (D) The proportion of B cells identified by flow cytometry reached 95.624%.

Supplementary figures
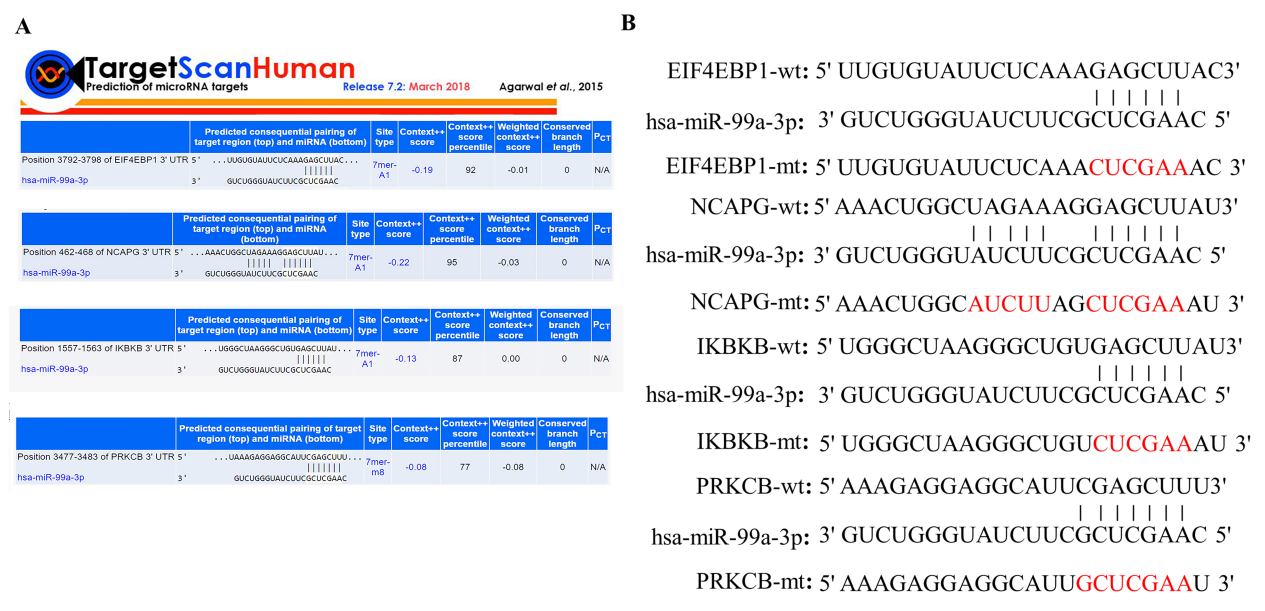
 2. Prediction of the binding site of miR-99a-3p and the target gene. (A, B) TargetScanHuman 7.2 was used to predict the binding sites of miR-99a-3p and the target genes EIF4EBP1, NCAPG, IKBKB, and PRKCB.


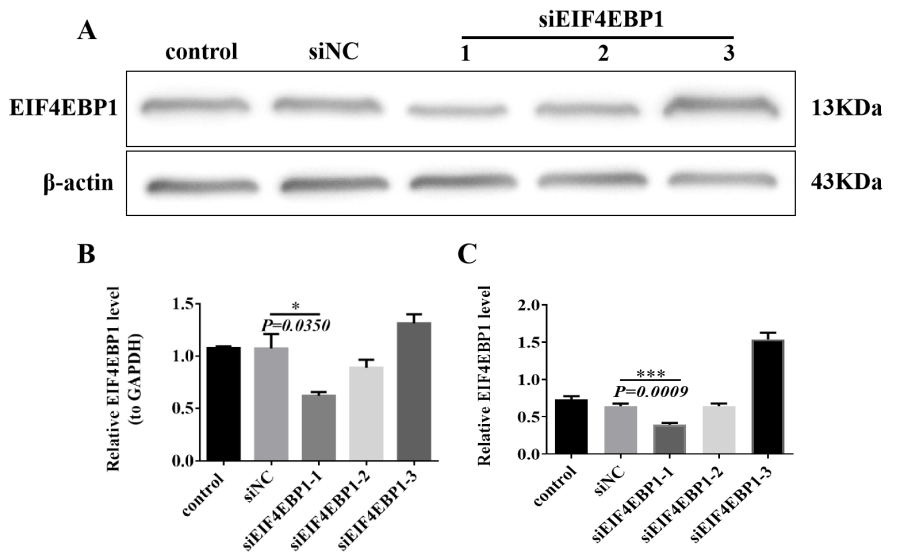


Supplementary figures 3. siEIF4EBP1 screening. (A) Western blotting detection of EIF4EBP1 expression in Ball-1 cells 48 h after transfection with control, siNC, siEIF4EBP1-1, siEIF4EBP1-2, and siEIF4EBP1-3. (B) RT-qPCR detection of EIF4EBP1 expression in Ball-1 cells 48 h after transfection with control, siNC, siEIF4EBP1-1, siEIF4EBP1-2, and siEIF4EBP1-3 (n=3). (C) Western blotting results showing EIF4EBP1 expression in Ball-1 cells after transfection (n=3).
